# Integrative Modeling of Accelerometry-Derived Sleep, Physical Activity, and Circadian Rhythm Domains With Current or Remitted Major Depression

**DOI:** 10.1001/jamapsychiatry.2024.1321

**Published:** 2024-06-12

**Authors:** Sun Jung Kang, Andrew Leroux, Wei Guo, Debangan Dey, Marie-Pierre F. Strippoli, Junrui Di, Julien Vaucher, Pedro Marques-Vidal, Peter Vollenweider, Martin Preisig, Kathleen R. Merikangas, Vadim Zipunnikov

**Affiliations:** 1 Genetic Epidemiology Research Branch, Intramural Research Program, National Institute of Mental Health, Bethesda, Maryland; 2 Department of Biostatistics and Informatics, University of Colorado, Anschutz Medical Campus, Aurora; 3 Psychiatric Epidemiology and Psychopathology Research Center, Department of Psychiatry, Lausanne University Hospital and University of Lausanne, Prilly, Switzerland; 4 Johns Hopkins Bloomberg School of Public Health, Baltimore, Maryland; 5 Service of Internal Medicine, Department of Medicine and Specialties, Fribourg Hospital and University of Fribourg, Switzerland; 6 Service of Internal Medicine, Department of Medicine, Lausanne University Hospital and University of Lausanne, Switzerland

## Abstract

**Importance:**

Accelerometry has been increasingly used as an objective index of sleep, physical activity, and circadian rhythms in people with mood disorders. However, most prior research has focused on sleep or physical activity alone without consideration of the strong within- and cross-domain intercorrelations; and few studies have distinguished between trait and state profiles of accelerometry domains in major depressive disorder (MDD).

**Objectives:**

To identify joint and individual components of the domains derived from accelerometry, including sleep, physical activity, and circadian rhythmicity using the Joint and Individual Variation Explained method (JIVE), a novel multimodal integrative dimension-reduction technique; and to examine associations between joint and individual components with current and remitted MDD.

**Design, Setting, and Participants:**

This cross-sectional study examined data from the second wave of a population cohort study from Lausanne, Switzerland. Participants included 2317 adults (1164 without MDD, 185 with current MDD, and 968 with remitted MDD) with accelerometry for at least 7 days. Statistical analysis was conducted from January 2021 to June 2023.

**Main Outcomes and Measures:**

Features derived from accelerometry for 14 days; current and remitted MDD. Logistic regression adjusted for age, sex, body mass index, and anxiety and substance use disorders.

**Results:**

Among 2317 adults included in the study, 1261 (54.42%) were female, and mean (SD) age was 61.79 (9.97) years. JIVE reduced 28 accelerometry features to 3 joint and 6 individual components (1 sleep, 2 physical activity, 3 circadian rhythms). Joint components explained 58.5%, 79.5%, 54.5% of the total variation in sleep, physical activity, and circadian rhythm domains, respectively. Both current and remitted depression were associated with the first 2 joint components that were distinguished by the salience of high-intensity physical activity and amplitude of circadian rhythm and timing of both sleep and physical activity, respectively. MDD had significantly weaker circadian rhythmicity.

**Conclusions and Relevance:**

Application of a novel multimodal dimension-reduction technique demonstrates the importance of joint influences of physical activity, circadian rhythms, and timing of both sleep and physical activity with MDD; dampened circadian rhythmicity may constitute a trait marker for MDD. This work illustrates the value of accelerometry as a potential biomarker for subtypes of depression and highlights the importance of consideration of the full 24-hour sleep-wake cycle in future studies.

## Key Points

**Question:**

What is the overlap between accelerometry-assessed sleep, physical activity, and circadian rhythms, and do they distinguish state and trait in major depressive disorder (MDD)?

**Findings:**

This cross-sectional study of 2317 adults used a multimodal data-reduction technique and found that joint components outweighed individual variation in the 3 domains. MDD was characterized by reduced and more fragmented physical activity, lower amplitude of circadian rhythm, and later timing of both sleep and the peak of physical activity.

**Meaning:**

These findings highlight the importance of characterizing 24-hour rhythms with accelerometry for future research and to define targets for interventions for MDD.

## Teaser

This cross-sectional study examines joint and individual components of the accelerometry-derived domains of sleep, physical activity, and circadian rhythms and assesses associations with major depressive disorder (MDD) among adults from Lausanne, Switzerland.

## Introduction

Accelerometry has been increasingly used as an objective index of sleep, physical activity, and circadian rhythms in people with mood disorders. Findings from more than 50 accelerometry-based studies of major depressive disorder (MDD) have converged in demonstrating lower and more variable daytime activity, poorer sleep efficiency, and dampened circadian rhythms in people with depression compared with controls.^1,2,3,4,5^ The consistency of these patterns illustrates the utility of accelerometry as an objective tool to characterize 24-hour rhythms that could inform etiology, interventions, and prevention of MDD.

There are several challenges to the interpretation of accelerometry findings that limit its clinical utility and scalability as an objective biomarker of MDD. First, many studies of accelerometry in MDD have been conducted on clinical or convenience samples of people during acute episodes of depression, often with less than 7 days of assessment. Second, most prior research has focused on sleep or physical activity alone without consideration of the strong within- and cross-domain intercorrelations. Third, few studies have distinguished between trait and state profiles of accelerometry domains in MDD that have different implications for interpretations of the findings. Those studies that have compared accelerometry features during acute and remitted episodes of MDD over 2 weeks or more have shown that alteration in physical activity and circadian rhythmicity may index trait markers for depression because of their persistence after remission and their familial specificity, By contrast, sleep difficulties are more likely to reflect current state because of their resolution after acute episodes of depression.^2,6,7,8,9^

With the growing application of research and commercial accelerometry devices in both clinical and community samples, the need for harmonization of the procedures and analytical methods for accelerometry data are increasingly apparent.^10^ Although the sleep and physical activity fields have begun to establish common procedures for administration and analysis of accelerometry data,^11,12^ integration of cross-study findings has been impeded by wide variation in the selection of features derived from accelerometry.^12^ Whereas most studies have focused on either sleep or physical activity alone,^3,5^ others have reported results from dozens of features without a priori hypotheses.^13^ Moreover, to our knowledge, the interdependence among the features included in these domains has not been considered in previous work on depression despite their derivation from a common underlying measure of acceleration.

Here, we use a technique termed “Joint and Individual Variation Explained” (JIVE), an integrative dimension-reduction technique for multimodal multivariate data, which yields estimates of the joint and individual variance explained by sleep, physical activity, and circadian rhythms, the 3 domains derived from accelerometry.^14^ We assessed the utility of JIVE as a method to quantify associations between current and remitted MDD with the joint and individual variation of the accelerometry-derived features in a large community-based cohort.^15,16^

## Methods

### Sample

The sample includes 2317 adults with accelerometry for at least 7 days in wave 2 (2014-2018) of the Colaus|PsyCoLaus study,^15^ a cohort study of adults age 35 to 75 years randomly selected from the general population of Lausanne, Switzerland.^16^ Rates of comorbid current anxiety disorders and substance use disorders and current antidepressant or benzodiazepine use are shown in Table 1. Participants with a lifetime history of bipolar disorder were excluded from these analyses. Self-reported ethnicity was collected for both the epidemiologic and genetic analyses. The study was approved by the Institutional Ethics Committee of the University of Lausanne, and written informed consent was obtained. This study followed the Strengthening the Reporting of Observational Studies in Epidemiology (STROBE) reporting guideline.

**Table 1.** Characteristics of Accelerometry-Derived Features of Sleep, Physical Activity, and Circadian Rhythms of the Colaus|PsyCoLaus Sample by Current and Remitted Major Depressive Disorder (MDD)

Characteristic	Mean (SD)			*P* value^a^
	No MDD (n = 1164)	Current MDD (n = 185)	Remitted MDD (n = 968)	
Sex, No. (%)				
Female	502 (43.13)	131 (70.81)	628 (64.88)	<.001
Male	662 (56.87)	54 (29.19)	340 (35.12)	
Race, No. (%)				
White	1086 (93.30)	167 (90.27)	893 (92.25)	.29
Other^b^	78 (6.70)	18 (9.73)	75 (7.75)	
Current anxiety^c^	33 (2.84)	21 (11.35)	57 (5.89)	<.001
Current SUD^d^	5 (0.43)	3 (1.62)	5 (0.52)^e^	.13
Current medication^f^	32 (2.75)	64 (34.59)	128 (13.22)	<.001
Age, y	63.39 (10.32)	58.66 (8.76)	60.47 (9.42)	<.001
BMI	26.30 (4.64)	26.75 (5.36)	26.34 (4.77)	.49
Accelerometry features				
Sleep				
Onset per 24 h	23.44 (1.16)	23.60 (1.30)	23.55 (1.13)	.046
Wakeup per 24 h	7.18 (1.11)	7.55 (1.34)	7.29 (1.06)	<.001
Duration, h	6.69 (1.01)	6.87 (1.13)	6.76 (0.97)	.03
Midpoint per 24 h	3.31 (1.01)	3.58 (1.17)	3.42 (0.97)	.001
Efficiency, %	0.87 (0.06)	0.87 (0.05)	0.88 (0.05)	<.001
NWB	2.08 (1.64)	2.17 (1.49)	1.84 (1.48)	.001
NSB	13.97 (3.63)	14.51 (3.59)	13.87 (3.66)	.09
Physical activity				
TAC	42 588 (13 655)	41 750 (12 374)	42 847 (12 577)	.57
TLAC	6733 (368.4)	6689 (378.8)	6741 (342.5)	.19
TST	1160 (109.7)	1155 (108.8)	1152 (101.3)	.26
LiPA	183.2 (61.27)	187.8 (62.49)	190.2 (57.71)	.03
MVPA	97.14 (65.65)	96.93 (59.91)	97.72 (57.34)	.97
SATP	0.07 (0.02)	0.07 (0.02)	0.07 (0.02)	.12
ASTP	0.25 (0.08)	0.25 (0.07)	0.24 (0.07)	.11
Circadian rhythms				
fPC1	−0.22 (10.49)	1.27 (10.80)	−0.30 (9.78)	.15
fPC2	0.54 (8.15)	−1.53 (8.64)	−0.52 (7.76)	<.001
fPC3	0.00 (5.89)	−0.05 (5.99)	0.05 (5.91)	.97
fPC4	−0.21 (3.92)	0.56 (4.05)	0.19 (3.90)	.01
RA	0.82 (0.07)	0.82 (0.06)	0.83 (0.06)	.11
IV	0.66 (0.13)	0.67 (0.14)	0.67 (0.13)	.91
IS	0.36 (0.10)	0.35 (0.09)	0.35 (0.09)	.16
Mesor	29.57 (9.48)	28.99 (8.59)	29.75 (8.73)	.57
Amp	24.48 (10.16)	23.84 (8.77)	24.32 (9.67)	.71
Acro	−3.65 (0.32)	−3.76 (0.34)	−3.71 (0.29)	<.001
L5	4.29 (1.30)	4.32 (1.36)	4.24 (1.26)	.63
M10	50.26 (17.38)	49.17 (15.16)	50.26 (15.92)	.69
L5Time	−0.15 (0.94)	0.11 (1.09)	−0.06 (0.93)	.001
M10Time	−0.12 (1.23)	0.32 (1.30)	0.08 (1.22)	<.001

Abbreviations: Acro, acrophase; Amp, amplitude; ASTP, active to sedentary transition probability; BMI, body mass index (calculated as weight in kilograms divided by height in meters squared); fPC, functional principal component; IS, interday stability; IV, intradaily variability; L5, acceleration value of the least active 5 hours; L5Time, central timing of least active 5 hours; LiPA, light intensity physical activity (duration in minutes); M10, acceleration value of the most active 10 hours; M10Time, central timing of most active 10 hours; Mesor, Midline Statistics of Rhythm; MVPA, moderate to vigorous physical activity (duration in minutes); NSB, number of blocks of night sleep within the sleep period time; NWB, number of times awake during the night for at least 5 minutes; RA, relative amplitude; SATP, sedentary to active transition probability; SUD, substance use disorder; TAC, total accelerometry count; TLAC, total log-transformed accelerometry count; TST, total sedentary time (duration in minutes); MDD, major depressive disorder.

^a^ *P* values from χ^2^ test for sex and analysis of variance tests for the rest of the variables.

^b^ As this is a Swiss dataset, they do not have additional information regarding the other ethnicity category.

^c^ Current anxiety disorders including agoraphobia, generalized anxiety disorder, panic disorder, and social anxiety disorder.

^d^ Current illicit drug (including cannabis) abuse or dependence.

^e^ Frequency missing = 2.

^f^ Current antidepressant and benzodiazepine use.

### Measures

#### Diagnosis of MDD

*Diagnostic and Statistical Manual of Mental Disorders* (Fourth Edition) criteria for MDD were ascertained with a clinically administered semistructured diagnostic interview for genetic studies (DIGS),^17^ which included the anxiety disorder sections (agoraphobia, generalized anxiety disorder, panic disorder, and social anxiety disorder) of the French version^18^ of the Schedule for Affective Disorders and Schizophrenia-Lifetime and Anxiety Disorder version (SADS-LA).^19^ Lifetime substance use disorders included alcohol and drug use disorders or dependence on cocaine, heroin, stimulants, sedatives, or hallucinogens.^20^

#### Accelerometry

Inclusion criteria for the accelerometry analyses required at least 7 valid days of actigraphy data (defined as >16 hours of estimated wear time). This yielded a total of 28 862 valid days in 2317 adults in these analyses. Features of sleep (SL), physical activity (PA), and circadian rhythms (CR) were derived from a wrist-worn triaxial accelerometer (GENEActiv [Activinsights Ltd]), a device that has been validated against reference methods.^21,22,23,24,25^ The devices were preprogrammed with a 50-Hz sampling frequency and placed on participants’ right wrists. Data were downloaded by GENEActiv software version 2.2 (Activinsights Ltd) and aggregated into 60-second epoch files. Sleep features were obtained from GGIR summary output files (R-package GGIR version 2.2-0),^26,27^ and the features of PA and CR were derived from the minute-level accelerometry count (R package mMARCH.AC^14,21^). Earlier analyses of these data show the specific details of the data processing and missing information in the Colaus Study.^28^

### Statistical Analysis

Most standard accelerometry-derived features of SL, PA, and CR were obtained for each day and averaged across valid days for each participant (eTable 1 in Supplement 1). The SL domain is characterized by measures of timing (Onset, Wakeup, Midpoint), duration, efficiency, and continuity and/or fragmentation (number of times awake during the night for at least 5 minutes (NWB), the number of blocks of night sleep within the sleep period (NSB). The PA domain is characterized by the total volume (TAC, TLAC), composition (TST, LiPA, MVPA), and fragmentation (SATP, ASTP). The CR domain is characterized by temporal/diurnal landmarks (fPC1-fPC2), measures of strength and related subcomponents (RA, L5 and M10, Mesor, Amplitude), variability (IV, IS), and timing (L5Time, M10Time, fPC3, fPC4, Acro).

JIVE performs integrative dimension-reduction of multiple features grouped within several different domains^29^ by incorporating the covariation of features within and across domains as a proxy for information generated by a group of latent variables. The eFigure in Supplement 1 presents the conceptualization of JIVE. Four groups of latent variables are derived from the SL, PA, and CR that explain (1) joint variation shared across SL, PA, and CR; and (2) individual variation specific to each domain of SL, PA, and CR. These latent variables are uncorrelated by construction and thus can be used simultaneously in regression analyses as novel measures that capture either joint or individual information from the 3 domains.^14^

Traditional regression analysis may result in unstable estimation if there is substantial redundancy or high levels of intercorrelations among the variables because it typically applies an additive model of the parameters of the 3 domains of SL, PA, and CR independently in a linear fashion (eg, Depression = SL + PA + CR)*.* JIVE-component regression addresses this redundancy in 2 steps: (1) 4 orthogonal latent variables (denoted as Joint, SL–individual, PA– individual, CR–individual) are derived such that SL = Joint + SL-individual, PA = Joint + PA-individual, CR = Joint + CR-individual; and (2) regression models the association between JIVE components with Depression (eg, Depression = Joint + SL-individual + PA-individual + CR-individual). This yields a more accurate interpretation of joint and individual associations between the outcome and core domains than traditional regression models that do not account for the cross-domain interrelationships. More technical details on JIVE can be found in eTable 1 in Supplement 1. Statistical analysis was performed using SAS software version 9.4 (SAS Institute) from January 2021 to July 2023.

## Results

### JIVE Structure

Among 2317 adults included in the study, the mean (SD) age at the time of accelerometer wear was 61.79 (9.97) years; 1261 participants (54.42%) were female; and the mean (SD) body mass index (BMI, calculated as weight in kilograms divided by height in meters squared) of 26.35 (4.75) (Table 1). The Colaus sample was primarily White (92.62%), with 7.38% from other ethnic backgrounds. The mean (SD) number of days with actigraphy data was 12.46 (1.34) with a range of 7 to 14 days. Approximately half of the participants met the criteria for at least 1 episode of MDD (49.76%), of which 41.78% were remitted, and 7.98% were current at the time of the assessment. Table 1 summarizes the demographic and clinical characteristics of the sample and the domain-specific features, including 7 features for SL, 7 features for PA, and 14 features for CR. JIVE estimated that the optimal number of components is 3 for the joint, 1 for SL, 2 for PA, and 3 for CR variations. Joint components accounted for 58.5% of the total variation in the SL domain, 79.5% of the total variation in the PA domain, and 54.5% of the total variation in the CR domain. These high levels of joint variation indicate the substantial overlap across these 3 domains. Individual components explained 34.4%, 17.6%, and 25.5% of the total variation in SL, PA, and CR domains, respectively. Only 7.1%, 2.9%, and 20.0% of the total variation in SL, PA, and CR, respectively, remained unexplained by the estimated JIVE model (Figure).

**Figure.**
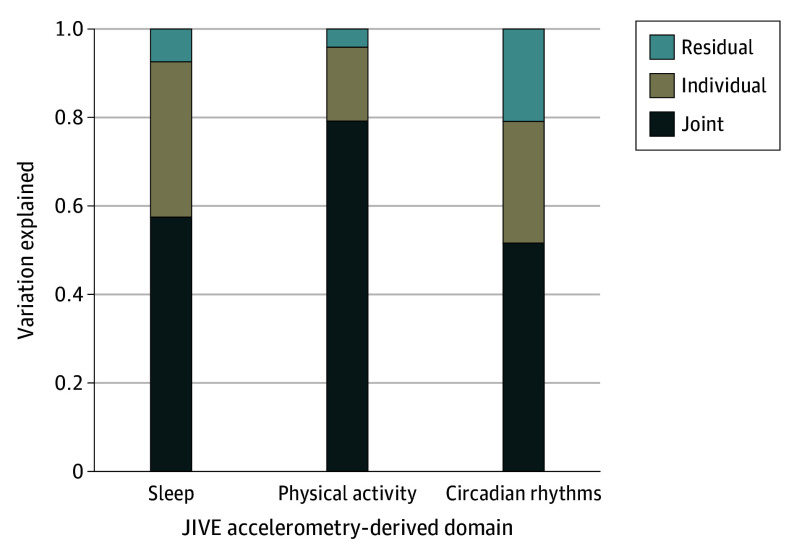
Joint and Individual Variation Explained (JIVE) by 3 Accelerometry-Derived Domains in the Colaus|PsyCoLaus Sample (N = 2317) Joint components of JIVE explained 58.5% of the total variation in the sleep domain, 79.5% in the physical activity domain, and 54.5% in the circadian rhythms domain. Individual components explained 34.4% of the total variation in the sleep domain, 17.6% in the physical activity domain, and 25.5% in the circadian rhythms domain. Residual (unexplained by JIVE) variation was 7.1%, 2.9%, and 20.0% of the total variation in sleep, physical activity, and circadian rhythms domains, respectively.

### Joint JIVE Components

eTable 2 in Supplement 1 shows the direction of the loadings (+/−) and squares of loadings that capture the relative magnitude of the estimated joint JIVE components. For each component, the squares of loadings add up to 1. Therefore, their magnitude can be interpreted as the proportional importance of the corresponding feature for that specific component. We focus on features with more than 5% of proportional variation. Participants with higher positive Joint-1 scores have higher total volume of PA (TAC, TLAC, fPC1) and time spent in both levels of intensity of PA (LiPA, MVPA), lower nonactive time (TST), higher average level of activity (Mesor), higher peak levels of activity (Amp), higher activity levels in the 5 least active hours (L5), less frequent transitions to sedentary behaviors (ASTP), and more frequent transitions to active bouts (SATP). Participants with higher positive Joint-2 scores have later timing of sleeping (Onset, Wakeup, Midpoint), later peak of rest-activity rhythm acrophase (Acro), later midpoints of both L5 and M10, and later peak of activity defined by fPC2. Participants with higher positive Joint-3 scores have shorter duration (measured both by Duration and the contrast of Wakeup and Onset) and lower sleep efficiency.

### Individual JIVE Components

eTable 3 in Supplement 1 shows the direction (+/−) and squares of loadings (to measure relative magnitude) for the estimated individual JIVE components as follows: JIVE-SL-1 loads positively on NWB, NSB and negatively on Efficiency; JIVE-PA-1 loads negatively on SATP, LiPA, TLAC, and positively on MVPA; JIVE-PA-2 loads positively on ASTP, SATP, TST, and negatively on LiPA; JIVE-CR-1 loads positively on IS and Acro and negatively on fPC3, fPC4, IV, and L5Time; JIVE-CR-2 loads positively on fPC3 and M10, and negatively on fPC4 and RA. Finally, JIVE-CR-3 loads positively on fPC3, fPC4, IS, and M10, and negatively on IV.

### Logistic Regression Analyses

Table 2 presents the results of the analyses of the JIVE scores by the 3 subgroups of MDD including lifetime, current, and remitted MDD, with sex, age, BMI, anxiety, and substance use disorders (SUD) as covariates. The findings indicated that individuals with either remitted or current MDD differed significantly from controls on the first and second joint JIVE scores. JIVE-Joint-1 scores, which were associated with higher amounts of and less fragmented physical activity, were negatively associated with MDD (*P* = .003), whereas JIVE-Joint-2 scores that were associated with later timing of both sleep and the peak of physical activity were positively associated with MDD (*P* = .01). Individual JIVE-PA-2 scores that reflected more frequent transitions between active and sedentary behaviors, less time in LiPA, and more time in sedentary and MVPA were negatively associated with all 3 subgroups of MDD. Individual JIVE-CR-1 scores that reflected earlier L5 time, later acrophase, and higher interday stability were negatively associated with lifetime and remitted MDD. Individual JIVE-CR-2 scores that reflected dampened circadian rhythm as measured by RA were positively associated with lifetime MDD, although the this result was not statistically significant when modeling current and remitted MDD separately. Post hoc analyses were conducted to examine adjustment for current medication use on these findings (eTable 4 in Supplement 1). The results were largely robust to adjustment for current medication use, with significant associations persisting for lifetime MDD models. However, joint components were no longer statistically significant when comparing participants with remitted MDD to controls. Additionally, JIVE-Joint-2 was no longer significantly different comparing current MDD with controls.

**Table 2.** Regression Models of Associations Between Major Depression Subgroups and JIVE Features With Adjustment for Correlates in the Colaus|PsyCoLaus Sample (N = 2317)^a^

Major depression, variable	Lifetime (n = 1153 vs 1164)		Current (n = 185 vs 1164)		Remitted (n = 968 vs 1164)	
	OR (95% CI)	*P* value	OR (95% CI)	*P* value	OR (95% CI)	*P* value
JIVE results						
JIVE-Joint-1	0.86 (0.78-0.95)	.003	0.72 (0.60-0.88)	.001	0.88 (0.80-0.98)	.02
JIVE-Joint-2	1.12 (1.02-1.22)	.01	1.19 (1.02-1.39)	.03	1.10 (1.00-1.20)	.046
JIVE-Joint-3	0.97 (0.88-1.06)	.44	0.94 (0.79-1.12)	.49	0.98 (0.89-1.08)	.66
JIVE-SL-1	0.97 (0.89-1.06)	.56	1.15 (0.97-1.37)	.12	0.95 (0.86-1.04)	.24
JIVE-PA-1	0.94 (0.85-1.03)	.16	0.98 (0.82-1.17)	.83	0.93 (0.85-1.02)	.14
JIVE-PA-2	0.84 (0.75-0.93)	.002	0.74 (0.60-0.91)	.005	0.86 (0.77-0.96)	.008
JIVE-CR-1	0.86 (0.78-0.96)	.007	0.83 (0.68-1.02)	.08	0.87 (0.78-0.97)	.01
JIVE-CR-2	1.11 (1.01-1.21)	.03	1.19 (1.00-1.43)	.06	1.09 (0.99-1.20)	.08
JIVE-CR-3	0.99 (0.90-1.09)	.91	1.10 (0.91-1.31)	.33	0.98 (0.89-1.09)	.75

Abbreviations: BMI, body mass index (calculated as weight in kilograms divided by height in meters squared); JIVE-CR-1, the first JIVE individual circadian rhythms score; JIVE-CR-2, the second JIVE individual circadian rhythms score; JIVE-CR-3, the third JIVE individual circadian rhythms score; JIVE-joint-1, the first JIVE joint score; JIVE-joint-2, the second JIVE joint score; JIVE-joint-3, the third JIVE joint score; JIVE-PA-1, the first JIVE individual physical activity score; JIVE-PA-2, the second JIVE individual physical activity score; JIVE-SL-1, the first JIVE individual sleep score; OR, odds ratio; SUD, substance use disorder.

^a^ Models are adjusted for covariates: age, sex, BMI, current anxiety, and current SUD.

## Discussion

Through the application of a novel integrative modeling technique to accelerometry data from a large population-based sample, our study’s results suggest the importance of simultaneous consideration of the 3 domains of SL, PA and CR extracted from accelerometry as an objective measure of 24-hour rhythms in MDD. The joint and individual features that distinguish MDD in general, as well as state and trait indices of depression, have important relevance for both the treatment and prevention of MDD. The associations between the amount of PA, the strength of CR, and the timing of sleep and the peak of physical activity across both current and remitted depression highlight their role as trait markers for MDD as well as potential targets for prevention of depressive episodes. The high magnitude of cross-domain variation that superseded that of any of the accelerometry-derived domains of SL, PA, and CR alone also highlights the importance of joint consideration of these domains in elucidating their associations with human health.

Application of JIVE to find the interdependencies among the 3 domains extracted from accelerometry demonstrates that the joint cross-domain variation supersedes that explained by any of these domains alone. Therefore, research focusing on any single domain alone may provide misleading interpretations of the associations between these domains with the disorders of interest. By examining identified components in those with remitted depression, we further explored the potential of these patterns to distinguish state from trait manifestations of MDD. This may have critical implications for translation into etiologic inference as well as interventions in SL, PA, and CR in people who have MDD.

The lack of associations between MDD and the sleep domain (except timing) supports the conclusions of prior research that sleep parameters are more likely to reflect current state rather than an enduring marker of MDD.^2,8,9^ The first 2 joint components that are more likely to represent trait markers for depression were primarily associated with the amount of PA, the strength of CR, and timing of both sleep and the peak of PA, along with other indices of CR. This extends the evidence regarding the importance of the timing of these domains as a core manifestation of MDD as previously shown for bipolar disorder.^30,31,32^

This work demonstrates the utility of accelerometry to identify the salient components of rest-activity patterns that may index trait markers for depression. The joint influences across SL, PA, and CR associated with MDD may reflect the underlying biologic and genetic factors that have been difficult to detect in studies that have sought to identify the genetic architecture of individual sleep or activity parameters such as self-reported usual sleep duration,^33^ morningness-eveningness,^34^ physical activity,^35^ chronotype, or accelerometer-derived features^36,37,38,39^ without simultaneous consideration of the other domains. Future studies should interrogate potential genetic, biological, and environmental factors that may underlie these joint latent factors in both population-based and clinical samples.

The shared influence of circadian rhythmicity with each of the 2 joint domains suggests that timing is a critical component of individualized interventions. For example, although sleep interventions have been effective in reducing insomnia,^40^ there has been increasing recognition of the importance of incorporating sleep timing into the treatment of sleep difficulties,^41^ as well as modifications of daytime activity patterns that may influence sleep. Likewise, the heterogeneity of the effects of physical activity interventions for depression^42^ could be enhanced by the incorporation of the timing of activity and sleep patterns as components of intervention programs.

### Limitations

This study has limitations. This work reflects our initial investigation of common variance underlying domains extracted from accelerometry, and several limitations should be considered in the interpretation of these findings. First, these data are limited to averages of accelerometry measures across 2 weeks that may not reflect enduring patterns of these domains. Although this is standard in the field, we are now repeating these assessments in this and other samples to characterize the stability of these patterns over time. Second, our interpretation of the findings that persist across remission as an index of trait markers for MDD. However, the patterns of sleep and physical activity herein could also reflect consequences of prior episodes of depression. Third, the findings may not generalize to different age, ethnic, or geographic subgroups. Despite the consistency in findings from accelerometry in mood disorders across adults, they may not extend to children and adolescents.^2^ Although this study is unique in examining accelerometry in a representative sample of the general population, the demographic characteristics of the sample may not generalize to different geographic and ethnic subgroups, as shown by recent studies in the US.^43^ Fourth, the findings may not apply to subgroups of depression such as the atypical or melancholic specifiers of MDD that we have examined previously.^28^ Fifth, we could not evaluate the potential effects of medications on patterns of activity identified in these analyses. Even though many prior studies of accelerometry in mood disorders have not adjusted for medications,^9^ our post hoc analyses that adjusted for medication use did yield some differences in the findings. Confounding between medication use and severity complicates the interpretation of these results. There is a need for systematic studies that test the effects of medications on objective measures of physical activity and sleep. For example, a recent study used accelerometry parameters to track symptom response in a placebo-controlled trial of sertraline for MDD, with inconclusive findings.^44^ Sixth, although we did control for comorbid anxiety and substance use disorders in these analyses, further exploration of differences in activity patterns among those with specific combinations and order of onset of comorbid conditions such as posttraumatic stress^45^ and physical conditions would be important in testing the specificity of these results. Seventh, the *P* values we report in this manuscript are not adjusted for multiple comparisons that could result in inflated type I error rates. However, through an ongoing international collaborative effort designed to harmonize mobile assessments of mood disorders, the mobile Motor Activity Research Consortium for Health (mMARCH) network, efforts are under way to replicate and extend these findings to broader diagnostic groups, younger and more diverse samples, and geographic settings.

Another limitation of these analyses is that the structure of the 3 domains considered in this study may not extend to subgroups examined in the second logistic regression step of our application of JIVE.^46^ JIVE enforces orthogonality constraints that makes interpretation of both directionality and composition of higher-order JIVE components and their joint associations with the outcome challenging. Future analyses will test a 1-step supervised JIVE (sJIVE) that simultaneously calculates JIVE components that are most associated with the continuous outcomes^47^ and extend this work to investigate the variability of these patterns. We also plan to exploit the rapid emergence of a new generation of fusion approaches that account for the distributional and temporally local distributional information that may reveal interdependencies between domains not captured by the original JIVE approach^48^ and the growing number of multimodal dimension-reduction techniques for mixed data types that can incorporate binary, ordinal, truncated, categorical, and other data types.^49^

## Conclusion

This cross-sectional study’s characterization of common manifestations of these regulatory systems is a first step in providing a more accurate depiction of the 24-hour sleep-wake cycle. The findings highlight the heuristic potential of future research that investigates the biologic systems that may underlie shared pathophysiologic mechanisms across multiple domains along with aggregate environmental influences on these systems such as light, temperature, and season. Closer approximation of these domains to those elucidated in basic circadian science may provide greater insight into the role of these homeostatic systems in mood disorders. This approach may also be extended to other health conditions such as cardiovascular disease, sleep disorders, and cognitive decline, for which accelerometry has been an important tool to track regulatory systems in the population.

## Notes

Supplement 1. **eFigure.** Conceptual Representation of JIVE Decomposition of Variation in Domains of Sleep, Physical Activity, and Circadian Rhythms **eTable 1.** Definitions and Abbreviations of Domains and Variables Extracted From Accelerometry **eTable 2.** JIVE Loading Squares by Domain and Variables in the Colaus|PsyCoLaus Sample. **eTable 3.** JIVE Individual Loading Squares in the Colaus|PsyCoLaus Sample **eTable 4**. Odds Ratio, Lower and Upper 95% Confidence Limits and P-Values From Regression Models of the Associations Between Major Depression Subgroups on JIVE Features With Adjustment for Correlates Including Current Medication in the Colaus|PsyCoLaus Sample (n=2317)

Supplement 2.
Data Sharing Statement
