# Supplementary material for: Integrative Modeling of Accelerometry-Derived Sleep, Physical Activity, and Circadian Rhythm Domains With Current or Remitted Major Depression
Source: JAMA Psychiatry. 2024 Jun 12;81(9):911–8. doi: 10.1001/jamapsychiatry.2024.1321 (PMC11170457; doi:10.1001/jamapsychiatry.2024.1321)
Supplement: Supplement 1. — eFigure. Conceptual Representation of JIVE Decomposition of Variation in Domains of Sleep, Physical Activity, and Circadian Rhythms eTable 1. Definitions and Abbreviations of Domains and Variables Extracted From Accelerometry eTable 2. JIVE Loading Squares by Domain and Variables in the Colaus|PsyCoLaus Sample. eTable 3. JIVE Individual Loading Squares in the Colaus|PsyCoLaus Sample eTable 4. Odds Ratio, Lower and Upper 95% Confidence Limits and P-Values From Regression Models of the Associations Between Major Depression Subgroups on JIVE Features With Adjustment for Correlates Including Current Medication in the Colaus|PsyCoLaus Sample (n=2317) [file jamapsychiatry-e241321-s001.pdf]

Supplemental Online Content

Kang SJ, Leroux A, Guo W, et al. Integrative modeling of accelerometry-derived sleep, physical activity, and circadian rhythm domains with current or remitted major depression. *JAMA Psychiatry*. Published online June 12, 2024. doi:10.1001/jamapsychiatry.2024.1321

eFigure. Conceptual Representation of JIVE Decomposition of Variation in Domains of Sleep, Physical Activity, and Circadian Rhythms
eTable 1. Definitions and Abbreviations of Domains and Variables Extracted From Accelerometry
eTable 2. JIVE Loading Squares by Domain and Variables in the Colaus|PsyCoLaus Sample
eTable 3. JIVE Individual Loading Squares in the Colaus|PsyCoLaus Sample
eTable 4. Odds Ratio, Lower and Upper 95% Confidence Limits and P-Values From Regression Models of the Associations Between Major Depression Subgroups on JIVE Features With Adjustment for Correlates Including Current Medication in the Colaus|PsyCoLaus Sample (n=2317)

This supplemental material has been provided by the authors to give readers additional information about their work.

© 2024 Kang SJ et al. *JAMA Psychiatry.*

eFigure. Conceptual representation of JIVE decomposition of variation in domains of sleep, physical activity, and circadian rhythms.

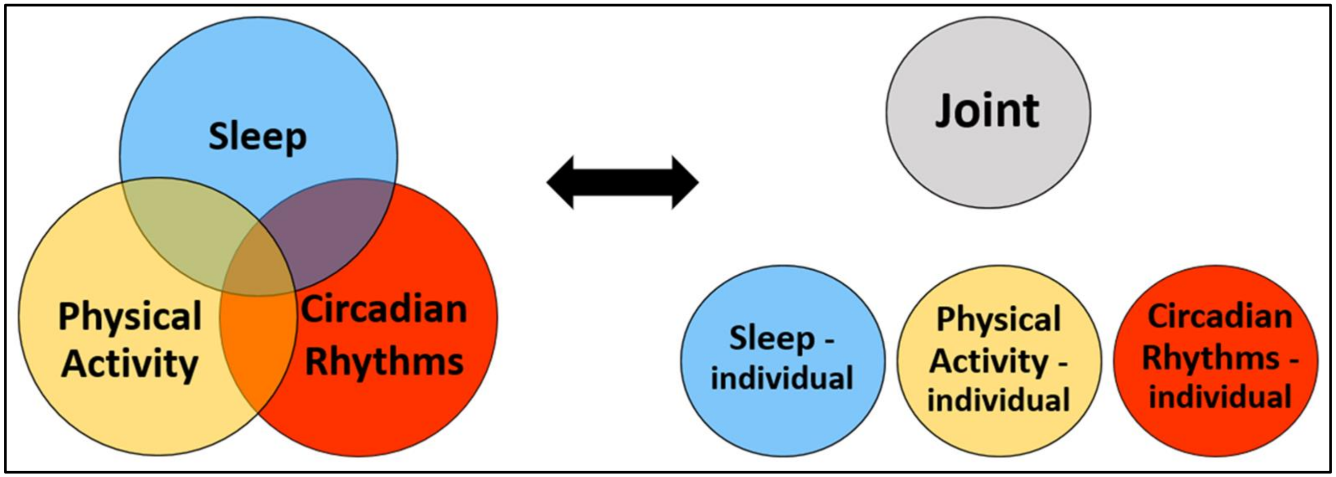

© 2024 Kang SJ et al. *JAMA Psychiatry.*

eTable 1. Definitions and abbreviations of domains and variables extracted from accelerometry

Domain	Variable	Description
**Sleep**	Onset	Detected onset of sleep since the midnight of the previous night
	Wakeup	Detected waking time (after sleep period) since the midnight of the previous night
	Midpoint	Sleep midpoint
	Duration	Sleep duration
	Efficiency	Sleep efficiency
	NSB	Number of night sleep block within the sleep period time window
	NWB	Number of times awake during the night for at least 5 min
**Physical Activity**	TAC	Total activity count
	TLAC	Total log-transformed activity count
	TST	Total sedentary time
	LiPA	Total light activity time (50 < AC < 100)
	MVPA	Total moderate to vigorous activity time (AC > 100)
	SATP	Sedentary to active transition probabilities (AC < 50)
	ASTP	Active to sedentary transition probabilities (AC > 50)
**Circadian Rhythms**	fPC1	1st principal component score from functional principal component analysis
	fPC2	2nd principal component score from functional principal component analysis
	fPC3	3rd principal component score from functional principal component analysis
	fPC4	4th principal component score from functional principal component analysis
	RA	Relative amplitude
	IV	Intra-daily variability
	IS	Inter-day stability
	Mesor	Midline-estimating statistic of rhythm, a rhythm adjusted mean
	Amp	Amplitude, a measure of variability around the mean
	Acro	Acrophase, a measure of timing of the peak value of cycle
	M10	Acceleration value of the most active 10 hours
	L5	Acceleration value of the least active 5 hours
	M10Time	Central timing (circular mean) of most active 10 hours
	L5Time	Central timing (circular mean) of least active 5 hours

© 2024 Kang SJ et al. *JAMA Psychiatry.*

Description of eTable1

Sleep domain includes measures of timing (Onset, Wakeup, Midpoint), sleep duration, sleep efficiency, and the number of times awake during the night for at least 5 minutes (NWB), the number of blocks of night sleep within the sleep period (NSB).

Physical Activity domains includes 1) Total Accelerometry Count (TAC), Total Log-Transformed Accelerometry Count (TLAC), and Total Sedentary Time (TST) as measures of total volume of physical activity; 2) total number of minutes spent in Light intensity Physical Activity (LiPA) and in Moderate to Vigorous Physical Activity (MVPA) as measures of PA composition; and 3) Active to Sedentary Transition Probability (ASTP) and Sedentary to Active Transition Probability (SATP) as measures of fragmentation of PA. PA features were calculated based on the minute-level accelerometry count data, which was the average of the twelve 5-second ENMO measures with a threshold of 50 for LiPA and 100 for MVPA.

Circadian Rhythms domain includes measures that reflect the timing of sleep and physical activity including the first four functional principal components (fPC1-fPC4) (which summarize daily-specific variational features such as overall daily activity level, early versus late morning activity, biphasic versus monophasic activity, and early versus late biphasic activity) [25], Relative Amplitude (RA); Intra-daily Variability (IV); M10; L5; M10Time; L5Time; three features derived from cosinor model including Midline Statistics of Rhythm (Mesor), Amplitude (Amp), Acrophase (Acro); and Inter-day Stability (IS) [14].

The choice of the first four fPCs is based on two factors. First, they capture most of the diurnal variability (77.2%) in our data. Second, choosing fPC1-fPC4 was based both on our own and other investigators experience in the application of functional principal component analysis (fPCA) in accelerometry-studies of diurnal rest-activity rhythms in large national cohorts such as National Health

© 2024 Kang SJ et al. *JAMA Psychiatry.*

and Nutrition Examination Survey (NHANES) and the UK biobank that focused primarily on the first four fPCs (Xiao et al., 2022, Stein et al., 2023). Additionally, fPCs beyond the 4th typically capture a relatively low proportion of variance in the data and their shapes tend to be very difficult to interpret due to their very "wiggly" shape.

Extra details on JIVE: JIVE applies the following steps: 1) multiple features are measured within each domain, resulting in the estimation of multiple latent joint and individual components; 2) certain joint components may capture joint variation in a subset of domains rather than all three domains (for example, physical activity and circadian rhythms); 3) similar to PCA, each participant receives a "score" for each latent variable, corresponding to how much variability in their features is explained by each latent variable. Interpretation of regression results depends on the "loadings" for each feature on the corresponding latent variable; and 4) the optimal number of joint and individual components are estimated, assuming that the optimal model does not capture all variability in the data.

Here, we applied the JIVE scores derived from the three domains to test the associations between these domains with trait and state MDD in a series of logistic regression models. During pre-processing, all features were normalized via a two-step procedure: (1) a normal-score transformation by applying a standard normal cumulative distribution function (CDF) to the inverse empirical CDF; and (2) a global normalization in each domain as in the original JIVE [14]. The optimal number of components was estimated using the "r.jive" R package [25]. JIVE scores were calculated, and the associations between the JIVE scores features in three domains and MDD were tested with logistic regression models that included sex, age, BMI, and comorbid anxiety and substance use disorders as covariates. Post hoc analyses were also conducted to adjust for current psychotropic medication use in the sample. Because JIVE

© 2024 Kang SJ et al. *JAMA Psychiatry.*

scores are uncorrelated, their inclusion in models does not induce the instability of high-intercorrelations in regression models. All JIVE analyses were performed using “r.jive” R package [25]. The regression analyses were performed using SAS software (version 9.4).

© 2024 Kang SJ et al. *JAMA Psychiatry.*

eTable 2. JIVE loading squares [1,2] by domain and variables in the Colaus|PsyCoLaus Sample (n=2317)

	Variable	JIVE-Joint-1		JIVE-Joint-2		JIVE-Joint-3	
Sleep	Onset	(-)	0.04%	**(+)**	**18.76%**	**(+)**	**9.09%**
	Wakeup	(-)	0.67%	**(+)**	**20.03%**	**(-)**	**7.11%**
	Midpoint	(-)	0.34%	**(+)**	**25.48%**	(+)	0.06%
	Duration	(-)	0.17%	(+)	0.00%	**(-)**	**44.69%**
	Efficiency	(+)	0.12%	(+)	0.07%	**(-)**	**9.77%**
	NWB	(-)	0.14%	(-)	0.03%	(+)	1.45%
	NSB	(+)	0.01%	(+)	0.00%	(-)	3.64%
	Total loading square in SL		1.48%		64.37%		75.80%
Physical Activity	TAC	**(+)**	**12.68%**	(+)	0.09%	(+)	0.03%
	TLAC	**(+)**	**10.37%**	(+)	0.15%	(+)	2.39%
	TST	**(-)**	**12.32%**	(-)	0.11%	(+)	0.06%
	LiPA	**(+)**	**8.04%**	(+)	0.11%	(-)	0.01%
	MVPA	**(+)**	**11.48%**	(+)	0.09%	(-)	0.17%
	SATP	**(+)**	**5.60%**	(+)	0.05%	(+)	2.77%
	ASTP	**(-)**	**9.59%**	(-)	0.10%	(+)	2.32%
	Total loading square in PA		70.07%		0.70%		7.73%
Circadian Rhythms	fPC1	**(-)**	**5.22%**	(+)	0.15%	(-)	0.12%
	fPC2	(-)	0.05%	**(-)**	**10.37%**	(-)	0.31%
	fPC3	(+)	0.01%	(-)	0.37%	**(+)**	**5.09%**
	fPC4	(+)	0.01%	(-)	0.21%	(-)	0.92%
	RA	(+)	3.41%	(+)	0.21%	(-)	2.48%
	IV	(-)	1.29%	(-)	0.13%	(+)	0.88%
	IS	(+)	0.19%	(-)	0.15%	(-)	0.98%
	Mesor	**(+)**	**6.34%**	(+)	0.05%	(+)	0.01%
	Amp	**(+)**	**5.30%**	(+)	0.07%	(-)	0.77%
	Acro	(+)	0.00%	**(-)**	**7.56%**	(-)	0.04%
	M10	(+)	0.24%	(-)	0.11%	(+)	4.66%
	L5	**(+)**	**6.12%**	(+)	0.05%	(-)	0.12%
	M10Time	(-)	0.24%	**(+)**	**8.78%**	(-)	0.08%
	L5Time	(-)	0.02%	**(+)**	**6.73%**	(-)	0.00%
	Total loading square in CR		28.44%		34.92%		16.46%

© 2024 Kang SJ et al. *JAMA Psychiatry.*

Abbreviations: NWB, number of times awake during the night for at least 5 minutes; NSB, the number of blocks of night sleep within the sleep period time; TAC, Total Accelerometry Count; TLAC, Total Log-Transformed Accelerometry Count; TST, Total Sedentary Time; LiPA, Light intensity Physical Activity; MVPA, Moderate to Vigorous Physical Activity; SATP, Sedentary to Active Transition Probability; ASTP, Active to Sedentary Transition Probability; fPC, functional principal component; RA, Relative Amplitude; IV, Intra-daily Variability; IS, Inter-day Stability; Mesor, Midline Statistics of Rhythm; Amp, Amplitude; Acro, Acrophase; M10, Acceleration value of the most active 10 hours; L5, Acceleration value of the least active 5 hours; M10Time, Central timing of most active 10 hours; L5Time, Central timing of least active 5 hours.

[1] The loading squares greater than 5% are in bold.

[2] (+)/(-) signs indicate the directions of joint loadings.

© 2024 Kang SJ et al. *JAMA Psychiatry.*

eTable 3. JIVE individual loading squares [1,2] in the Colaus|PsyCoLaus Sample (n=2317)

Sleep			Physical Activity					Circadian Rhythms						
Variable	JIVE-SL-1		Variable	JIVE-PA-1		JIVE-PA-2		Variable	JIVE-CR-1		JIVE-CR-2		JIVE-CR-3	
Onset	(-)	2.67%	TAC	(+)	0.05%	(+)	2.51%	fPC1	(-)	0.42%	(+)	1.19%	(-)	0.47%
Wakeup	(+)	0.97%	TLAC	**(-)**	**7.22%**	(+)	2.91%	fPC2	(+)	2.73%	(+)	0.01%	(+)	0.09%
Midpoint	(-)	0.16%	TST	(+)	1.75%	**(+)**	**5.35%**	fPC3	**(-)**	**8.41%**	**(+)**	**17.41%**	**(+)**	**7.28%**
Duration	(+)	0.08%	LiPA	**(-)**	**27.59%**	**(-)**	**35.93%**	fPC4	**(-)**	**29.74%**	**(-)**	**14.85%**	**(+)**	**21.03%**
Efficiency	**(-)**	**29.42%**	MVPA	**(+)**	**7.18%**	(+)	3.28%	RA	(+)	0.58%	**(-)**	**18.74%**	(-)	2.79%
NWB	**(+)**	**36.91%**	SATP	**(-)**	**52.48%**	**(+)**	**10.05%**	IV	**(-)**	**6.52%**	(+)	2.68%	**(-)**	**32.67%**
NSB	**(+)**	**29.79%**	ASTP	(-)	3.73%	**(+)**	**39.96%**	IS	**(+)**	**14.10%**	(-)	3.06%	**(+)**	**25.52%**
								Mesor	(+)	0.00%	(-)	0.12%	(-)	0.03%
								Amp	(+)	0.32%	(-)	2.56%	(+)	0.27%
								Acro	**(+)**	**17.61%**	(+)	1.43%	(+)	0.08%
								M10	(-)	0.15%	**(+)**	**36.07%**	**(+)**	**9.69%**
								L5	(+)	0.12%	(-)	0.71%	(+)	0.02%
								M10Time	(-)	0.02%	(+)	0.24%	(+)	0.05%
								L5Time	**(-)**	**19.28%**	(-)	0.95%	(+)	0.03%

Abbreviations: NWB, number of times awake during the night for at least 5 minutes; NSB, the number of blocks of night sleep within the sleep period time; TAC, Total Accelerometry Count; TLAC, Total Log-Transformed Accelerometry Count; TST, Total Sedentary Time; LiPA, Light intensity Physical Activity; MVPA, Moderate to Vigorous Physical Activity; SATP, Sedentary to Active Transition Probability; ASTP, Active to Sedentary Transition Probability; fPC, functional principal component; RA, Relative Amplitude; IV, Intra-daily Variability; IS, Inter-day Stability; Mesor, Midline Statistics of Rhythm; Amp, Amplitude; Acro, Acrophase; M10, Acceleration value of the most active 10 hours; L5, Acceleration value of the least active 5 hours; M10Time, Central timing of most active 10 hours; L5Time, Central timing of least active 5 hours.

[1] The loading squares greater than 5% are in bold.

[2] (+)/(-) signs indicate the directions of joint loadings.

© 2024 Kang SJ et al. *JAMA Psychiatry.*

eTable 4. Odds ratio, lower and upper 95% confidence limits and p-values[1] from regression models of the associations between Major Depression Subgroups on JIVE Features with adjustment for correlates including current medication in the Colaus|PsyCoLaus Sample (n=2317)

**Major Depression**	**Lifetime (n=1153 vs.1164)**				**Current (n=185 vs. 1164)**				**Remitted (n=968 vs. 1164)**			
Variable	OR	L 95	U 95	*P*	OR	L 95	U 95	*P*	OR	L 95	U 95	*P*
JIVE-Joint-1	0.89	0.80	0.98	**0.019**	0.76	0.61	0.94	**0.010**	0.91	0.82	1.01	0.071
JIVE-Joint-2	1.10	1.00	1.20	**0.039**	1.15	0.97	1.36	0.113	1.09	0.99	1.19	0.085
JIVE-Joint-3	1.00	0.91	1.10	0.989	1.01	0.84	1.23	0.881	1.00	0.91	1.11	0.953
JIVE-SL-1	0.95	0.87	1.05	0.321	1.09	0.90	1.32	0.365	0.93	0.85	1.02	0.135
JIVE-PA-1	0.92	0.84	1.01	0.094	0.93	0.77	1.14	0.492	0.92	0.84	1.01	0.093
JIVE-PA-2	0.84	0.76	0.94	**0.003**	0.78	0.62	0.98	**0.033**	0.86	0.76	0.96	**0.008**
JIVE-CR-1	0.87	0.79	0.97	**0.015**	0.88	0.71	1.10	0.273	0.87	0.78	0.98	**0.017**
JIVE-CR-2	1.10	1.00	1.20	**0.045**	1.20	0.98	1.45	0.073	1.08	0.98	1.19	0.124
JIVE-CR-3	0.97	0.88	1.07	0.560	1.04	0.85	1.27	0.733	0.97	0.87	1.07	0.493

Abbreviations: OR, Odds Ratio; L 95, Lower 95% Confidence Limit; U 95, Upper 95% Confidence Limit; SUD, substance use disorder; JIVE-joint-1, the first JIVE joint score; JIVE-joint-2, the second JIVE joint score; JIVE-joint-3, the third JIVE joint score; JIVE-SL-1, the first JIVE individual sleep score; JIVE-PA-1, the first JIVE individual physical activity score; JIVE-PA-2, the second JIVE individual physical activity score; JIVE-CR-1, the first JIVE individual circadian rhythms score; JIVE-CR-2, the second JIVE individual circadian rhythms score; JIVE-CR-3, the third JIVE individual circadian rhythms score.

[1] Statistically significant result for JIVE components are in bold.

[2] The model is adjusted for covariates: Sex, Age, BMI, Current Anxiety, Current SUD and Current Medication.

© 2024 Kang SJ et al. *JAMA Psychiatry.*
