# Supplementary material for: Integrative Modeling of Accelerometry-Derived Sleep, Physical Activity, and Circadian Rhythm Domains With Current or Remitted Major Depression
Source: JAMA Psychiatry. 2024 Jun 12;81(9):911–8. doi: 10.1001/jamapsychiatry.2024.1321 (PMC11170457; doi:10.1001/jamapsychiatry.2024.1321)
Supplement: Supplement 2. — Data Sharing Statement [file jamapsychiatry-e241321-s002.pdf]

Data Sharing Statement

Kang. Integrative Modeling of Accelerometry-Derived Sleep, Physical Activity, and Circadian Rhythm Domains With Current or Remitted Major Depression. *JAMA Psychiatry*. Published June 12, 2024. doi:10.1001/jamapsychiatry.2024.1321

Data
Data available: Yes
Data types: Deidentified participant data
How to access data: martin.preisig@chuv.ch; kathleen.merikangas@nih.gov
When available: With publication

Supporting Documents
Document types: Statistical/analytic code
How to access documents: ksj1011@gmail.com
When available: With publication

Additional Information
Who can access the data: researchers whose proposed use of the data has been approved
Types of analyses: for a specified purpose
Mechanisms of data availability: after approval of a proposal and with a signed data access agreement
